# Age-Friendly Environments for Healthy Ageing in Place: Refinement of a Multidimensional Index and Regional Disparities in South Korea

**DOI:** 10.3390/healthcare14162651

**Published:** 2026-08-21

**Authors:** Miri Kim

**Affiliations:** Department of Social Welfare, Chungju Campus, Korea National University of Transportation, Chungju-si 27469, Republic of Korea; mirikim@ut.ac.kr

**Keywords:** age-friendly environment, ageing in place, healthy ageing, environmental gerontology, regional disparities, South Korea

## Abstract

**Highlights:**

**What are the main findings?**

- This study empirically evaluated and refined an Age-Friendly City Index as a district-level measure of environments supporting healthy ageing-in-place in South Korea.
- Exploratory comparisons showed that non-metropolitan municipalities had higher observed index scores than metropolitan municipalities across all dimensions, with the largest standardized difference in the physical environment.

**What are the implications of the main findings?**

- The refined Index provides a preliminary diagnostic framework for benchmarking age-friendly environments and informing healthy ageing-in-place policies.
- Findings highlight the need for region-specific public health and urban planning strategies to reduce inequalities in age-friendly environments.

**Abstract:**

Background/Objectives: South Korea faces one of the world’s most rapid demographic transitions. As population ageing accelerates, creating environments that support healthy ageing-in-place has become a critical public health priority. Grounded in environmental gerontology and the WHO Age-Friendly Cities framework, Ko and colleagues developed the Age-Friendly City Index to assess physical, social, and service environments influencing older adults’ health and well-being; however, the index had only undergone face validity testing. This study empirically evaluated and refined the Index at the district level and examined regional disparities in age-friendliness across South Korean municipalities. Methods: Data from 89 randomly selected municipal (si-gun-gu) districts across 7 metropolitan and 8 provincial cities were drawn from 2020 national public administrative sources. Confirmatory factor analysis was conducted using Mplus 7.0. The retained indicators were standardized and aggregated into dimension and total scores. Exploratory comparisons between metropolitan and non-metropolitan municipalities were conducted using Welch’s *t*-tests. Results: The refined Index retained ten indicators across the three dimensions, including four for the physical environment, three for the social environment, and three for the service environment. The physical environment showed the strongest empirical coherence, whereas the social and service environments were weakly defined. Non-metropolitan municipalities had higher observed scores across all three dimensions and in the total score. Effect sizes ranged from moderate to large, with the largest difference observed in the physical environment. Conclusions: The refined Index offers a preliminary diagnostic framework for benchmarking age-friendliness, with the physical environment score providing the most dependable basis for comparison. Findings reveal pronounced regional disparities in per-older-resident environmental provision and underscore the need for region-specific public health and urban planning strategies to foster inclusive, supportive, and health-promoting communities for older adults.

## 1. Introduction

Population ageing has become one of the most widely recognized public health and social policy challenges worldwide. As life expectancy increases and fertility rates decline, societies are required to ensure the health, autonomy, functional ability and quality of life of older adults. South Korea, in particular, is experiencing one of the most rapid demographic transitions in the world. The proportion of adults aged 65 and over reached 20% in 2025, and is expected to increase sharply over the coming decades [1]. By 2070, adults aged 65 and over are projected to account for approximately 47.5% of the total population [1]. This demographic shift places substantial pressure on society as a whole, requiring major adjustments across multiple sectors, including healthcare, social welfare, housing, transportation, and community infrastructure. In this context, creating age-friendly environments that support ageing-in-place has become a critical public health priority, as such environments enable older adults to maintain health, functional ability, social connectedness, and independence within their communities.

The World Health Organization’s (WHO) Age-Friendly Cities and Communities framework provides a useful foundation for understanding how environments can promote active and healthy ageing-in-place [2]. Ageing-in-place refers to the ability of older adults to continue living safely, independently, and comfortably in their own homes and communities, despite changes in health or functional capacity [3]. As people age and their health declines, their dependence on neighborhood resources and community infrastructure increases, while their ability to move about, engage socially, and carry out everyday activities becomes progressively more constrained [4]. Therefore, the environment plays an increasingly important role in either supporting or limiting older adults’ daily functioning, participation, and health in later life [5,6]. In this regard, the physical, social, and service environments emphasized in the WHO framework are central to supporting continued independence, social participation, access to health-promoting resources, and well-being in later life [2,7].

In South Korea, supporting ageing-in-place has become a central policy commitment. According to the 2020 National Survey of Older Koreans, 88.6% of older adults reported that they wished to continue living in their own homes [8]. In response, the government introduced the Integrated Community Care policy in 2018 as a pilot initiative [9]. This policy was later institutionalized through the Act on Integrated Support for Community Care and expanded to nationwide implementation across all si-gun-gu districts from March 2026 [10]. In addition, the national age-friendly city designation system was introduced following amendments to the Welfare of Senior Citizens Act in 2026, strengthening the role of municipal governments in creating supportive environments for older residents [11]. These policy developments indicate a shift toward locating responsibility for age-friendly environments at the local level. However, South Korean municipalities differ substantially in population ageing, service infrastructure, and administrative capacity. This variation highlights the need for objective and comparable tools that can assess age-friendliness at the district level and identify communities where environmental support for older adults remains insufficient.

Despite growing policy and academic interest in age-friendly environments, measuring age-friendliness remains challenging. Existing measurement efforts have advanced the field, but many rely on older adults’ subjective perceptions or focus on selected aspects of the neighborhood environment, such as walkability, accessibility, or service availability. For example, one of the most widely adopted tools, the Age-Friendly Cities and Communities Questionnaire (AFCCQ), has been translated across multiple national contexts [12,13]. Although such perception-based instruments provide important insights into older adults’ lived experiences, their reliance on subjective assessments limits the ability to generate standardized and comparable indicators across administrative units. Objective district-level indicators are therefore needed to identify regional disparities in age-friendly environments and to provide local governments with evidence for prioritizing areas that require targeted improvement.

As in other countries, research in South Korea has often relied on older adults’ subjective assessments of local environments or on selected indicators of community resources. Ko and colleagues addressed this limitation by developing the Age-Friendly City Index for South Korean municipalities [14]. Using public administrative data, the Index assesses age-friendliness at the si-gun-gu district level and provides a standardized basis for comparing local environments. Consistent with the WHO Age-Friendly Cities and Communities framework [2], it organizes indicators into three environmental dimensions of physical, social, and service environments, capturing local conditions related to mobility, safety, social participation, access to health and welfare resources, and ageing-in-place.

However, the empirical validity of the Age-Friendly City Index by Ko et al. [14] has not been sufficiently established, as its development relied primarily on conceptual alignment and face validity. Although face validity is an important first step, it does not determine whether selected indicators reliably represent the dimensions they are intended to measure or contribute meaningfully to the assessment of age-friendliness [15]. This is especially important for policy-oriented indices, which must accurately identify whether communities provide the environmental conditions and health-promoting resources needed to support older adults [16]. Empirical examination is therefore needed to determine whether the Index can serve as a reliable tool for assessing local age-friendliness and guiding evidence-based policy planning.

Accordingly, this study aims to empirically examine and refine the Age-Friendly City Index at the municipal district level in South Korea. Using public administrative data from si-gun-gu districts, this study examines whether the indicators proposed in the Index adequately reflect the physical, social, and service dimensions of age-friendly environments. After refining the measurement structure through CFA, the study further assesses regional disparities in age-friendliness across municipalities. In doing so, this study contributes to the literature on age-friendly environments by moving beyond conceptual index development and providing empirical evidence for the construct validity of a district-level age-friendliness measure. A study by Kim et al. [17], which applied confirmatory factor analysis (CFA) and structural equation modeling to the AARP Age-Friendly Community Survey and demonstrated a multidimensional structure of perceived age-friendliness associated with older adults’ self-rated health, is closely related to the current study. However, their study validated a measure based on older adults’ subjective perceptions of their communities. The present study extends this line of research by empirically validating an age-friendly environment index constructed from objective public administrative data.

This study is expected to make several contributions. First, it offers a refined measurement tool that can be used to benchmark age-friendly environments across local communities. Second, it identifies which dimensions of age-friendliness show greater regional variation, thereby providing evidence for region-specific public health and urban planning strategies that target the environmental conditions most consequential for health. Third, although the analysis is based on South Korean municipalities, the approach has broader relevance for ageing societies worldwide. South Korea’s rapid demographic transition offers an early case for examining policy and public health challenges that other countries may soon face, and validating an administrative-data-based index provides a transferable approach for monitoring regional inequalities in age-friendly environments. Ultimately, the refined Age-Friendly City Index can support policymakers, researchers, and practitioners in designing more equitable and health-promoting communities for an ageing society.

## 2. Methods

### 2.1. Data

This study used a cross-sectional design to empirically examine and refine an index of district-level age-friendliness in South Korea. Age-friendliness was assessed using the Age-Friendly City Index developed by Ko et al. [14], which measures the age-friendliness of local communities at the si-gun-gu (city, county, and district) level using national public administrative data. In South Korea, si-gun-gu represents the basic local administrative unit at which many public services, welfare programs, transportation systems, and community infrastructure are planned and managed. The study examined 89 si-gun-gu districts randomly sampled from districts nationwide, in proportion to the size of the older population within each provincial-level division. This sampling strategy was used to reflect the geographic distribution of older adults across South Korea while including municipalities with different demographic and infrastructural characteristics. The resulting 89 districts covered 7 metropolitan cities and 8 provincial areas, including both urban and rural municipalities. This allowed the study to capture variation in age-friendly environments across diverse local contexts, ranging from highly urbanized districts to less densely populated rural communities. Regarding sample-size adequacy, Wolf et al. [18] reported that a one-factor CFA with four indicators required approximately 90 observations when the standardized factor loadings were 0.65, although the required sample size varied according to the magnitude of the loadings and other model characteristics. Because the dimensions in the present study were examined separately using relatively simple one-factor models, the sample of 89 districts was considered to provide a reasonable basis for preliminary CFA estimation.

The Age-Friendly City Index by Ko et al. [14] assesses the age-friendliness of local communities by measuring the availability and provision of community resources in relation to the size of the older population within each district. Consistent with the WHO Age-Friendly Cities and Communities framework, the Index organizes indicators into three dimensions of physical, social, and service environments, each comprising several sub-domains. First, the physical environment comprised three topic areas of outdoor spaces and buildings, transportation, and housing. This dimension was measured using indicators related to outdoor safety, accessibility, mobility, and residential stability, including CCTV cameras, safety emergency bells, air quality, security lights, silver zones, park area, heat-wave shelters, public toilets, bus and subway stops, road-crossing safety, residential welfare facilities, and rental housing, each calculated in relation to the older population where applicable. Second, the social environment comprised topic areas of social participation, social integration, and education and informatization. The dimension was measured using indicators that reflect opportunities for older adults’ participation and inclusion within the community, including leisure welfare facilities, traditional markets, organizations providing employment programs for older adults, free food service centers, lifelong educational institutions, education programs for older adults, and participation in lifelong education programs, each expressed relative to the number of older residents in the district. Third, the service environment comprised health care and welfare services, and included indicators of health, welfare, and care resources available to older adults, including hospital beds, doctors, medical welfare facilities, in-home welfare facilities, and care service facilities for older adults, calculated relative to the older population in each district. Data for the indicators were obtained from 2020 national public administrative sources. The full set of indicators, their data sources, and their computation formulas is summarized in Table 1.

### 2.2. Data Analysis

The study proceeded in four stages. First, the indicators of the original Index were reviewed and modified according to the availability of comparable administrative data at the district level and their conceptual alignment with each dimension. Second, the modified indicators were compiled from 2020 national administrative sources and screened. Third, the dimensional structure of the modified Index was examined through CFA, and indicators that did not perform adequately within their intended dimension were removed to obtain a refined measurement model. Fourth, the retained indicators were standardized and aggregated into dimension and total age-friendliness scores, which were then used to examine regional disparities across districts. All analyses were conducted using SPSS 18.0 for data preparation and descriptive statistics, and Mplus 7.0 for CFA. The following sections describe each analytic step in detail.

#### 2.2.1. Review and Modification of the Index Indicators

The original Age-Friendly City Index proposed by Ko et al. [14] comprised indicators across the physical, social, and service environments. Before constructing the district-level Index, each indicator was reviewed for the availability of comparable administrative data at the si-gun-gu level and for conceptual alignment, and several indicators were substituted or removed accordingly. The full composition of the original and modified indicators is presented in Table 1.

#### 2.2.2. Data Preparation

Prior to analysis, each indicator was screened for outliers using SPSS. For every indicator, a boxplot was generated, and values falling beyond Q3 + 1.5 × IQR or below Q1 − 1.5 × IQR were treated as outliers. Identified outliers were replaced with the nearest non-outlying value (the cutoff value) rather than deleted, in order to retain all 89 districts while limiting the influence of extreme observations. Missing data in the confirmatory factor analyses were handled using full-information maximum likelihood (FIML) estimation under the robust maximum likelihood estimator. Descriptive statistics, including means, standard deviations, skewness, and kurtosis, were computed to characterize the distributions of the indicators, with skewness within ±2 and kurtosis within ±7 taken as consistent with approximate normality.

#### 2.2.3. Confirmatory Factor Analysis

CFA was conducted to refine the dimensional structure of the modified Index and to identify indicators that did not perform adequately within their intended dimension. Analyses were performed separately for the physical, social, and service dimensions using Mplus 7.0.

Indicator refinement was guided by both theoretical considerations and statistical evidence. Standardized factor loadings were treated as diagnostic information and evaluated in relation to each indicator’s conceptual relevance, content coverage, and coding direction, consistent with recommendations that empirical performance be considered together with content validity and theoretical interpretability [17,19,20,21,22]. All indicators were coded before the analysis so that higher values theoretically represented greater age-friendliness.

Indicators with negative loadings were first examined for possible heterogeneity. However, the direction of a loading alone was not considered sufficient grounds for reverse coding or exclusion. Following this conceptual review, the models were re-estimated, and standardized loadings below 0.30 were used as an empirical indication that an indicator shared limited variance with the other indicators in the intended dimension [19,20]. The refinement process was iterative, with decisions based on both empirical performance and conceptual coherence. Exclusion from a reflective dimension score does not mean that the corresponding resource is unimportant to age-friendliness. It indicates that the indicator did not function consistently as a reflection of the same latent construct in the present sample.

Model fit was evaluated using the Comparative Fit Index (CFI ≥ 0.95), the Standardized Root Mean Square Residual (SRMR ≤ 0.08), and the Root Mean Square Error of Approximation (RMSEA ≤ 0.08) as criteria for good fit [23,24]. The chi-square statistic was reported but not used as a primary criterion, given its known sensitivity to sample size [25]. For just-identified models (df = 0), overall fit indices yield mathematically perfect values and therefore cannot provide evidence of model fit. These values were reported for transparency, while model evaluation focused on the sign, magnitude, and statistical significance of the standardized factor loadings, the explained variance of the indicators, and conceptual coherence. In addition, for each final measurement model, composite reliability (CR) and average variance extracted (AVE) were calculated to assess construct reliability and convergent validity. Values of CR ≥ 0.60 and AVE ≥ 0.50 were considered as conventional benchmarks [26,27].

For the final measurement models, localized model fit was examined using modification indices, standardized residuals, and residual correlations. Modification indices greater than 3.84, corresponding to the critical value of $\chi^{2}$ with one degree of freedom at α=0.05, were considered potentially indicative of localized model misspecification [28]. Modification indices were treated as diagnostic information and were interpreted in conjunction with the magnitude of the residuals and theoretical plausibility. These diagnostics were interpreted only for overidentified models because, in just-identified models (df=0), the observed covariance matrix is reproduced exactly and residual-based diagnostics and modification indices do not provide meaningful additional evidence of model adequacy [29].

#### 2.2.4. Construction of District-Level Age-Friendliness Scores

After the final set of indicators was established for each dimension, the retained indicators were converted to standardized scores to allow comparison and summation across indicators measured on different scales. Following the procedure used in the original Index, each indicator was transformed into a t-score (M = 50, SD = 10), computed as t=10∗z+50. For each district, a dimension score was calculated by averaging the t-scores of the indicators retained within that dimension, and a total age-friendliness score was obtained by summing the scores of three dimensions. Higher scores indicate greater age-friendliness. These district-level scores were then used to describe regional variation and to examine disparities across the physical, social, and service dimensions.

## 3. Results

### 3.1. Modification of the Index Indicators

The indicators of the original Age-Friendly City Index were reviewed in terms of district-level data availability and conceptual alignment with the intended dimension, prior to conducting the confirmatory factor analyses. This review resulted in several modifications to the original Index. The retained, replaced, and removed indicators are summarized in Table 1.

In the physical environment dimension, three modifications were made. First, the original indicator of street lights was replaced with security lights because comparable district-level data on street lights were difficult to obtain. Security lights were considered a conceptually appropriate substitute because they improve visibility in dark areas and facilitate safe movement and walking after dark for older adults. Second, shade screens were replaced with designated heat-wave shelters due to limitations in obtaining comparable district-level data on shade screens. Although shade screens are typically installed near pedestrian areas, such as crosswalks, to provide temporary shade while walking, heat-wave shelters capture a broader form of community-level heat protection. Heat-wave shelters in Korea are designated spaces where residents, including older adults who may not have adequate cooling at home, can rest and cool down during periods of extreme heat. Third, the transportation indicator was expanded by adding subway stops to bus stops. This modification was made because the dominant mode of public transportation may differ across districts, with some areas relying primarily on buses and others having more developed subway systems. Combining bus and subway stops therefore provides a more comprehensive measure of local public transportation accessibility.

In the social environment dimension, one modification was made. The original indicator of senior stores was replaced with the number of organizations participating in employment programs for older adults. Senior stores are one specific type of initiative within the nationally supported older adult employment program and therefore capture only a limited, store-based form of participation. By contrast, organizations participating in senior employment programs reflect a broader range of implementing organizations and participation opportunities for older adults. This replacement was therefore considered more appropriate for assessing the social integration dimension of the Index, as it better captures the availability of productive and social participation opportunities within the community.

In the service environment dimension, five modifications were made. First, the number of hospitals was replaced with the number of hospital beds, because hospital beds more directly reflect institutional medical capacity than the count of hospitals alone. Next, four indicators from the original Index were removed, which included subjective health status, health check-up rate, strength and flexibility exercise rate, and the rate of no suicidal ideation. These indicators reflect individual-level health status or health behavior rather than district-level environmental conditions. Because the present study aimed to construct an administrative-data-based measure of local age-friendliness at the district level, these person-attribute indicators were excluded from the modified Index.

In calculating the Index, two indicators were directionally adjusted so that higher values consistently indicated greater age-friendliness. Air quality was calculated as the inverse of the mean fine-dust concentration, and the older-adult traffic-accident indicator was reverse-scored to reflect road-crossing safety.

### 3.2. Refinement of the Age-Friendly City Index Through Confirmatory Factor Analysis

Each dimension of the Index was examined using a single-factor CFA, and indicators were removed sequentially. Indicators with negative standardized loadings were removed first, followed by indicators with standardized loadings below 0.30.

The physical dimension proceeded through three steps in this manner. For the social dimension, however, retaining the borderline indicators in an intermediate model produced an improper solution, which is a Heywood case with a negative residual variance. Therefore, the negative and below-threshold indicators were removed together in a single step. For the service environment dimension, hospital beds and the number of doctors showed the strongest loadings in the initial model but represented general, all-age medical infrastructure rather than welfare and care services specifically relevant to older adults. These two indicators were therefore removed on conceptual grounds, and the three older adult-specific welfare indicators were retained.

Table 2 summarizes the model fit statistics for each refinement step across the three dimensions, and Table 3 presents the unstandardized and standardized factor loadings for the final measurement model of each dimension. The standardized loadings at every refinement step are provided in Appendix A Table A1. More detailed results for each dimension are presented below.

#### 3.2.1. Physical Environment

The initial model for the physical environment included all twelve indicators and showed poor fit, χ**^2^**(54) = 111.747, *p* < 0.001, RMSEA = 0.110, CFI = 0.584, SRMR = 0.114. Two indicators, police-linked safety bells (β = −0.340) and public and private rental housing (β = −0.367), loaded negatively on the latent factor. This indicates that these indicators moved in the opposite direction from the other physical environment indicators. Because a higher provision of these resources would be expected to contribute positively to age-friendliness, their negative loadings suggested that they did not reliably reflect the same underlying dimension. These indicators were therefore removed in the first refinement step.

The resulting ten-indicator model showed improved fit, but the fit indices remained below conventional thresholds, χ**^2^**(35) = 59.452, *p* = 0.006, RMSEA = 0.089, CFI = 0.728, SRMR = 0.096. In this model, several indicators showed standardized loadings below 0.30. These included CCTV cameras (β = 0.221), air quality (β = 0.264), silver zones (β = 0.014), parks (β = 0.202), bus and subway stops (β = 0.125), and residential welfare facilities (β = 0.219). Because these indicators contributed little to the common physical environment dimension, they were removed in the next refinement step.

The final four-indicator model retained security lights (β = 0.758), heat-wave shelters (β = 0.768), public toilets (β = 0.476), and road-crossing safety (β = 0.575). This model fit the data well, χ**^2^**(2) = 0.041, *p* = 0.980, RMSEA = 0.000, CFI = 1.000, SRMR = 0.004. All four retained indicators loaded positively on the latent factor, with R**^2^** values ranging from 0.23 to 0.59. Together, these indicators represent a coherent dimension of outdoor safety and accessibility within the physical environment.

Residual diagnostics for the final physical environment model did not identify localized areas of misfit. All residual correlations were at or below ∣0.008∣, all standardized residuals were below ∣0.07∣, and no modification index exceeded 3.84 [28].

#### 3.2.2. Social Environment

The initial model for the social environment included all seven indicators and showed poor fit, χ**^2^**(14) = 57.812, *p* < 0.001, RMSEA = 0.189, CFI = 0.637, SRMR = 0.124. Three indicators loaded negatively on the latent factor: lifelong education institutions (β = −0.275), education programs for older adults (β = −0.881), and lifelong education participants (β = −0.922). In addition, free meal service centers showed a loading close to zero (β = 0.009). The negative loadings indicated that these indicators moved in the opposite direction from the other social environment indicators, despite being expected to contribute positively to age-friendliness. The near-zero loading for free meal service centers indicated that this indicator contributed little shared variance to the latent factor.

For this dimension, removing the negatively loading indicators and the below-threshold indicator in two separate steps was not feasible. An intermediate four-indicator model produced an improper solution, specifically a Heywood case with a negative residual variance for one indicator, and model fit statistics could not be computed. Therefore, the negatively loading and below-threshold indicators were removed together in a single refinement step, retaining the three indicators with adequate positive loadings.

The final three-indicator model retained leisure welfare facilities (β = 0.425), traditional markets (β = 0.356), and organizations providing employment programs for older adults (β = 0.616). The model was saturated (χ**^2^**(0) = 0.000, RMSEA = 0.000, CFI = 1.000, SRMR = 0.000). These values were mathematical consequences of exact identification and were not interpreted as evidence of model fit. All three indicators loaded positively and significantly on the latent factor (all *p* < 0.05), although their R**^2^** values of 0.13–0.38 indicated relatively limited shared variance. Residual diagnostics and modification indices were not interpreted for the final social environment models because the model was just-identified (df=0).

#### 3.2.3. Service Environment

The initial five-indicator model for the service environment showed acceptable overall fit, χ**^2^**(5) = 6.328, *p* = 0.275, RMSEA = 0.055, CFI = 0.983, SRMR = 0.048. However, the standardized loadings showed inconsistent directions across indicators. Hospital beds (β = 0.934) and doctors (β = 0.762) loaded strongly and positively on the latent factor, whereas the indicators reflecting older adult-specific welfare and care services loaded negatively or weakly. These included medical welfare facilities (β = −0.373), in-home welfare facilities (β = −0.052), and care service facilities (β = −0.525).

Hospital beds and doctors represented general healthcare capacity, whereas medical welfare facilities, in-home welfare facilities, and care-service facilities represented resources more directly aligned with older adults’ long-term care, home-based support, and continued living in the community [2,7,30,31]. The opposing loading patterns suggested that the original service dimension combined two conceptually distinct domains. Accordingly, the three older-adult-specific indicators were retained to define a narrower service dimension focused on welfare and care resources that support ageing in place.

In the final three-indicator model, all retained indicators loaded in a positive direction: medical welfare facilities (β = 0.370), in-home welfare facilities (β = 0.423), and care service facilities (β = 0.419). The final three-indicator service model was saturated (χ**^2^**(0) = 0.000, RMSEA = 0.000, CFI = 1.000, SRMR = 0.000). These values were not interpreted as evidence of model fit because they resulted from exact identification. The retained indicators represented a conceptually coherent dimension focused on older adult-specific welfare and care resources; however, their R**^2^** values of 0.14–0.18 indicated limited shared variance. Residual diagnostics and modification indices were not interpreted for the final service environment models because the model was just-identified (df=0).

#### 3.2.4. Final Refined Measurement Structure

The refinement process resulted in a final Index comprising a total of ten indicators. Four indicators were retained in the physical environment, three in the social environment, and three in the service environment. All retained indicators loaded positively on their respective latent factors (Refer to Table 3). The three dimensions differed in the extent to which their indicators shared common variance. The physical environment formed the most empirically cohesive dimension, with all four indicators loading significantly (*p* < 0.001) and R**^2^** values reaching 0.590. The social and service environments were more weakly defined, with lower loadings and smaller R**^2^** values (0.127–0.379 and 0.137–0.179, respectively). The service-environment loadings, in particular, did not reach conventional significance. This suggests that, although all three dimensions are interpretable, the physical environment indicators co-occur across districts far more consistently than the social and service indicators, which represent more heterogeneous sets of community resources. The physical environment dimension showed a CR of 0.745 and an AVE of 0.430. The social dimension yielded a CR of 0.458 and an AVE of 0.229, while the service dimension showed a CR of 0.369 and an AVE of 0.164.

### 3.3. Descriptive Characteristics of Age-Friendliness Scores

#### 3.3.1. Overall Distribution of Scores

Table 4 summarizes the district-level age-friendliness scores across the 89 districts. Each dimension score was computed as the mean of the t-scored indicators retained in the final measurement model, and the total age-friendliness score was the sum of the three dimensions scores. Because each indicator was standardized to a t-score, the three dimension scores each had a mean of approximately 50, and the total score had a mean of 150.0 (SD = 17.6), ranging from 124.3 to 193.8. Among the three dimensions, the physical environment showed the largest variation across districts (SD = 7.5), followed by the social (SD = 6.9) and service (SD = 6.6) environments.

#### 3.3.2. Disparities Across Districts

The total age-friendliness scores revealed substantial disparities across districts, ranging from 124.3 to 193.8. This represents a difference of nearly 70 points, equivalent to approximately four standard deviations. The lowest-scoring district was Seocho-gu in Seoul (124.3), whereas the highest-scoring district was Yeonggwang-gun in Jeollanam-do (193.8). More broadly, the lowest-scoring districts were concentrated in metropolitan Seoul, including Seocho-gu, Eunpyeong-gu, Nowon-gu, and Seongbuk-gu. In contrast, the highest-scoring districts were predominantly non-metropolitan counties (gun) in provincial areas, including Yeonggwang-gun, Jeungpyeong-gun, Wanju-gun, and Geochang-gun.

This pattern reflects the relative, per-older-population basis of the Index. Because each indicator was calculated relative to the size of the older population in each district, rural districts tended to score higher when they had a greater availability of facilities or resources per older resident. By contrast, densely populated metropolitan districts, even when they had substantial infrastructure in absolute terms, often showed lower scores because those resources were distributed across much larger older populations. In other words, the Index captures the availability of age-friendly resources per older resident rather than the absolute quantity of resources. As a result, metropolitan districts with abundant infrastructure may still appear less age-friendly on a per capita basis. The full district-level scores, including the number and proportion of older adults in each district, are provided in Appendix A Table A2.

To further examine the practical significance of these regional differences, exploratory comparisons were conducted between metropolitan and non-metropolitan municipalities (Table 5). Non-metropolitan municipalities had significantly higher observed index scores across all three dimensions. The largest difference was found for the physical environment, with non-metropolitan municipalities scoring 8.2 points higher than metropolitan municipalities (95% CI [5.7, 10.7], t = 6.54, *p* < 0.001, d = 1.31). Differences were also observed for the social environment (mean difference = 3.9, 95% CI [1.2, 6.6], t = 2.91, *p* = 0.005, d = 0.59) and the service environment (mean difference = 6.4, 95% CI [3.9, 8.8], t = 5.14, *p* < 0.001, d = 1.08). Consequently, the total age-friendliness score was 18.5 points higher in non-metropolitan municipalities (95% CI [12.5, 24.6], t = 6.07, *p* < 0.001, d = 1.23). The effect sizes ranged from moderate to large and were particularly pronounced for the physical and service environments.

## 4. Discussion

This study empirically refined the Age-Friendly City Index developed by Ko et al. [14] at the municipal district level and examined regional disparities in age-friendliness across South Korean municipalities. Although the original Index was conceptually grounded in the WHO Age-Friendly Cities and Communities framework, the findings showed that not all indicators functioned as empirically valid measures of their intended dimensions. Through CFA, the Index was refined into a final structure consisting of ten indicators. There were four indicators for the physical environment, three for the social environment, and three for the service environment. Among the three dimensions, the physical environment was the most empirically cohesive, whereas the social and service environments were more weakly defined. The overall model-fit indices of the final social and service models do not provide evidence of model fit because both models were saturated. Their measurement quality should therefore be evaluated using other sources of evidence, including factor loadings, explained variance, and conceptual coherence. The results also showed substantial regional disparities in age-friendliness, with the physical environment showing the greatest variation across districts. These findings suggest that policy-oriented indices require empirical examination to determine whether selected indicators meaningfully represent the environmental dimensions they are intended to measure. The refined Index offers a more parsimonious and empirically grounded tool for benchmarking local age-friendliness and identifying regional inequalities in environmental support for healthy ageing-in-place.

One of the main contributions of this study is that it moves beyond face validity and provides empirical evidence for the structure of an administrative-data-based age-friendly environment index. The CFA results showed that indicators that appear conceptually relevant to age-friendliness do not necessarily function as coherent empirical measures of the same underlying construct. This finding is particularly important for policy-oriented indices, because such tools are often used to compare municipalities, identify disadvantaged areas, monitor policy progress, and guide resource allocation [16]. For an index to be useful in these contexts, its indicators must operate consistently within the intended construct [15]. If indicators within the same dimension capture different or weakly related phenomena, the resulting dimension score may be difficult to interpret. By retaining indicators that showed conceptual relevance and empirical coherence, the refined Index provides a more reliable basis for benchmarking local age-friendliness and identifying regional inequalities in environmental support for healthy ageing-in-place.

First, the physical environment emerged as the most clearly defined dimension. The retained indicators include security lights, heat-wave shelters, public toilets, and road-crossing safety. They are all closely related to older adults’ ability to move safely and comfortably within their communities. Outdoor walking is one of the most common forms of physical activity in later life, and previous studies have linked neighborhood walkability and built-environment features to higher physical activity, better physical functioning, and improved physical and mental health among older adults [32,33].

Specifically, adequate outdoor lighting improves visibility and reduces the fall risk and fear that discourage older adults from venturing out after dark, helping preserve the independent outdoor mobility on which ageing-in-place depends [34,35]. Similarly, public toilets and safe road-crossing conditions reduce practical barriers to leaving the home. When public toilets are limited in availability or unevenly distributed, older adults may limit the distance or duration of their trips because of concerns about toileting needs [36]. Adequate public toilet availability can therefore expand the range of places older adults feel able to access and the amount of time they can comfortably remain outside the home. Safe road-crossing conditions are also important because older adults may experience slower walking speed, reduced balance, or delayed reaction time, which can increase both actual and perceived risks when navigating traffic environments [37]. By extending the distance, duration, and perceived safety of out-of-home movement, these resources support the everyday neighborhood engagement that ageing-in-place entails.

Heat-wave shelters support healthy ageing-in-place through a different mechanism. Rather than primarily facilitating movement, they function as protective community resources that provide safe cooling spaces for older adults during periods of extreme heat, particularly for those who lack adequate cooling at home. This is especially important because older adults are more susceptible to heat-related illness and mortality due to reduced thermoregulatory capacity, chronic health conditions, and diminished physiological resilience [38,39]. Evidence from South Korea further supports the relevance of this indicator. Kim et al. [40], using data from 119 urban administrative districts, found that higher levels of social isolation among older adults were associated with greater vulnerability to heatwave-related mortality. This suggests that health risk during extreme weather conditions is shaped not only by climatic exposure, but also by social and residential vulnerability. In this regard, community-based heat-wave shelters may have broader implications beyond physical protection from heat. As shared neighborhood spaces, they can also create opportunities for social contact, informal interaction, and connection with local support systems during periods of environmental risk. This is particularly relevant to healthy ageing-in-place because remaining in the community requires not only a safe home environment, but also access to nearby places where older adults can receive protection, maintain social connection, and avoid isolation during emergencies.

Taken together, the inclusion of these four indicators suggests that the physical environment dimension can be understood through two interrelated pathways. Security lights, public toilets, and safe road-crossing conditions support healthy ageing-in-place by reducing barriers to outdoor mobility and enabling older adults to use neighborhood spaces more safely and comfortably. Heat-wave shelters, meanwhile, support healthy ageing-in-place by protecting older adults from climate-related health risks and potentially strengthening social connection during periods of environmental vulnerability.

This finding is also consistent with environmental gerontology and person–environment fit perspectives. These perspectives emphasize that older adults’ behavior and well-being are shaped by the interaction between personal competence and environmental demands, and that the surrounding environment becomes increasingly important as health and functional capacity decline in later life [5,6]. As physical, sensory, or functional capacities decline with age, having supportive environmental facilities can help older adults continue to move through, use, and remain connected to their communities.

Second, the social environment was represented by leisure welfare facilities, traditional markets, and organizations participating in employment programs for older adults. These retained indicators further extend the interpretation of the physical environment dimension. As discussed above, neighborhood environments support healthy ageing-in-place not only by reducing barriers to outdoor mobility, but also by creating conditions through which older adults can remain connected to others during periods of vulnerability. The social environment dimension makes this relation more explicit by identifying the community settings where neighborhood access can be translated into sustained participation, routine interaction, and role continuity.

In this sense, the social dimension of age-friendliness reflects more than the presence of facilities. It captures the social infrastructure that allows older adults to remain embedded in everyday community life. Leisure welfare facilities provide formal community-based spaces where older adults can participate in organized programs, receive information, and interact with peers. Traditional markets serve as familiar everyday settings where older adults may maintain routine social contact, encounter neighbors, and remain connected to local community life [41]. Organizations participating in employment programs for older adults further extend the social environment by providing opportunities for productive engagement, social contribution, and role continuity after retirement. These resources are therefore relevant to healthy ageing-in-place because they support not only social contact, but also identity, purpose, and a sense of being needed within the community.

This also suggests that social age-friendliness can operate through multiple pathways. Leisure welfare facilities primarily represent formal and program-based participation. Traditional markets represent informal and routine neighborhood interaction. Employment-related organizations represent productive and role-based engagement. Together, these indicators show that healthy ageing-in-place requires environments where older adults can continue to participate in ordinary routines, exchange information and support, maintain familiar relationships, and preserve valued social roles. Such continuity of everyday social life is important for health because social participation and connectedness are closely linked to psychological well-being, reduced isolation, and sustained functioning in later life [42,43].

At the same time, the relatively lower shared variance among the social indicators indicates that social age-friendliness is a more heterogeneous construct than the physical environment. This does not necessarily weaken the relevance of the social dimension. Rather, it suggests that different forms of social infrastructure may be distributed and organized through different local mechanisms. For example, leisure welfare facilities may reflect welfare service provision, traditional markets may reflect local commercial and neighborhood history, and employment program organizations may reflect local labor-market opportunities and welfare administration. These resources may all contribute to social participation and connectedness, but they do not necessarily co-occur in the same districts.

From a policy perspective, this lack of co-occurrence implies that a district performing well in one form of social infrastructure may still have deficits in others. For example, a municipality may have sufficient leisure welfare facilities but fewer employment-related organizations. Relying only on an aggregate social environment score could therefore obscure more specific gaps in the types of social support available to older adults. This reinforces the value of a multi-indicator index, which can identify not only whether a district is generally more or less age-friendly, but also which particular forms of social infrastructure are insufficient. Such information is important for designing targeted interventions that strengthen the specific social resources needed to support healthy ageing-in-place.

Third, the service environment was represented by medical welfare facilities, in-home welfare facilities, and care-service facilities for older adults. This dimension requires particularly careful interpretation. In the initial model, hospital beds and doctors showed strong positive loadings, indicating that general medical infrastructure formed a coherent district-level construct. However, these indicators may reflect overall healthcare capacity available to the general population rather than welfare and care resources specifically designed to support older adults. Their removal was therefore conceptually justified, even though they performed strongly in statistical terms.

The final service environment dimension retained indicators that more directly correspond to the goal of supporting ageing-in-place. Medical welfare facilities, in-home welfare facilities, and care-service facilities for older adults reflect community-based resources that address the care and support needs that often emerge as health and functional capacity decline in later life. Unlike general medical infrastructure, these resources are more closely related to the practical conditions that enable older adults to continue living in familiar community settings, including access to long-term care, assistance with daily activities, home-based support, and welfare services tailored to older adults.

This distinction is important because healthy ageing-in-place cannot be reduced to the availability of hospitals or physicians alone. Older adults with frailty, chronic illness, functional limitations, or complex care needs often require continuous support that bridges medical treatment, long-term care, welfare services, daily living assistance, and protection of dignity [30]. In some cases, severely ill older adults whose needs are not adequately addressed by acute-care systems alone may come to rely on long-term care, welfare, or community-based care facilities for ongoing support. This illustrates how reliance on general medical infrastructure alone can leave important gaps in support for the most vulnerable older adults.

A recent case in South Korea illustrates how such reliance can expose gaps that general indicators of medical capacity do not capture. A severely ill woman in her eighties, who reportedly could not secure hospital admission and was instead cared for in a medical welfare facility, underwent a leg amputation there, after which the limb was mishandled during disposal [44]. Although the immediate controversy concerned waste management, the case also drew public attention to the situation of highly vulnerable older adults whose complex medical and care needs are met outside the acute-care system. Such situations underscore the importance of service environments specifically designed to respond to the complex and changing needs of older adults in long-term and community-based care settings. They also show that the service environment relevant to ageing-in-place is not simply a matter of how many hospital beds or doctors are available, but whether communities have older adult-specific care infrastructures capable of responding to those needs.

The retained service indicators therefore shift the focus from general healthcare capacity to older adult-specific care infrastructure. In-home welfare facilities are especially relevant because they can help older adults receive support while remaining in their own homes, thereby delaying or preventing unnecessary institutionalization [31]. Medical welfare facilities and care-service facilities also represent local care resources that can support older adults when home-based support is insufficient or when more intensive care is required within the community. Together, these indicators capture the service foundation needed to help older adults manage health-related challenges, maintain daily functioning, and continue living safely and with dignity in familiar community environments.

The refinement process should be interpreted with care. Sequential indicator removal informed partly by sample statistics may capitalize on sample-specific relationships [22]. In this study, factor loadings were evaluated together with conceptual relevance and content coverage, consistent with theory-informed refinement procedures [13,17,19,20,21,22]. Hospital beds and doctors were excluded despite their high loadings because they represented general healthcare capacity, whereas the retained indicators were more directly aligned with older adult-specific welfare, long-term care, and community-based support for ageing in place [2,7,30,31]. Because the same 89 districts were used for both refinement and evaluation, the resulting ten-indicator structure should be considered provisional and tested using independent data before being applied for policy benchmarking.

The substantial reduction in the number of indicators also narrowed the conceptual coverage of the Index. The refined structure places greater emphasis on indicators that showed both conceptual relevance and empirical coherence within the present sample, but it does not represent the full range of environmental features included in the original Index. Indicators excluded from the CFA-based structure may still capture important complementary aspects of age-friendliness. The refined Index should therefore be interpreted as a focused diagnostic framework for the retained physical, social, and service dimensions, not as a comprehensive measure of all aspects of age-friendly environments.

Beyond the interpretation of each dimension, the findings revealed substantial regional disparities in age-friendliness across municipalities. Exploratory group comparisons showed that non-metropolitan municipalities had higher observed scores than metropolitan municipalities across all three dimensions and in the total score. The standardized differences were large for the physical environment, service environment, and total age-friendliness score, and moderate for the social environment. Nevertheless, these differences should be interpreted with care because they largely reflect the relative, per-older-population basis of the Index. The scores represent the availability of resources in relation to the size of the older population and do not directly measure the absolute volume, geographic accessibility, quality, or actual utilization of local resources. Rural and other non-metropolitan municipalities with smaller older populations may therefore achieve relatively high scores despite having modest absolute facility counts, whereas metropolitan municipalities may receive lower scores because their resources serve substantially larger older populations. Accordingly, the observed effect sizes should not be interpreted as evidence that non-metropolitan municipalities necessarily provide better overall environments for older adults or that metropolitan municipalities have an absolute shortage of infrastructure. Comparisons across municipalities should therefore be interpreted together with absolute resource levels, demographic characteristics, population density, transportation conditions, and spatial accessibility. Given these interpretive limitations, the refined Index is best used as a diagnostic framework for identifying relative strengths and weaknesses across dimensions, rather than as a definitive ranking of municipalities.

Building on this understanding, the findings have important policy implications for healthy ageing-in-place. First, the refined Index can serve as a preliminary diagnostic framework for local governments. This is particularly relevant in South Korea, where integrated community care has been implemented nationwide and the age-friendly city designation system has become institutionalized since 2026 [10,11]. However, despite these recent policy developments, a standardized national indicator system for assessing local age-friendly environments has not yet been sufficiently established. The refined Index addresses this gap by providing empirically refined indicators across physical, social, and service environments, offering municipalities objective and comparable evidence for diagnosing local strengths and weaknesses and informing age-friendly policy planning. In addition, the usefulness of the refined Index extends beyond South Korea. Other ageing societies also face the challenge of assessing whether local environments provide the conditions necessary for ageing-in-place. Although the specific indicators may need to be adapted to each country’s policy context, administrative data system, and local service structure, the overall approach provides a transferable framework for monitoring age-friendly environments, comparing municipalities, and identifying regional inequalities in support for healthy ageing-in-place.

Second, the findings highlight the need for region-specific public health and urban planning strategies. The large differences between metropolitan and non-metropolitan municipalities in the physical and service environment scores indicate substantial regional disparities in resource availability relative to the size of the older population. Because the Index provides separate scores for the physical, social, and service environments, it can help municipalities identify which dimensions of age-friendliness require priority attention. In metropolitan municipalities, lower observed scores may signal the need to assess whether existing resources are sufficient and equitably distributed in relation to the number and needs of older residents. Municipalities with weaker physical environment scores may require priority investments in neighborhood-level resources such as outdoor lighting, safe road-crossing conditions, public toilets, and heat-wave shelters [34,35,36,37,38,39,40], whereas weaker service environment scores may indicate a need to strengthen community-based welfare and care resources for older adults [30,31]. Such investments should be understood not merely as urban amenities or service provision, but as components of public health infrastructure because they support older adults’ safe mobility, social participation, functional independence, and ability to continue living in their communities [30,31,32,33,34,35,36,42,43].

At the same time, policy responses should be tailored to local contexts rather than applied uniformly across all municipalities. In densely populated metropolitan districts, the key policy challenge may be to ensure that existing resources are sufficient, reachable, and equitably distributed relative to the size and needs of the older population. In rural or less densely populated districts, higher per capita resource availability does not necessarily guarantee practical access, service quality, or actual use, particularly when transportation barriers, long travel distances, or limited service capacity remain. Therefore, local governments should examine not only whether resources exist, but also whether older adults can reach them, use them, and benefit from them in everyday life.

Overall, the refined Index should be used as a diagnostic framework for identifying dimension-specific and indicator-specific gaps. Such use can help municipalities move beyond general assessments of age-friendliness and develop targeted strategies that respond to their own environmental deficits. By guiding locally tailored investments in physical infrastructure, social participation resources, and older adult-specific care services, the refined Index can support efforts to reduce inequalities in age-friendly environments and strengthen the local conditions that make healthy ageing-in-place possible.

This study has several limitations that should be acknowledged. First, the analysis was based on a cross-sectional design using administrative data from a single year. Longitudinal applications of the Index would allow researchers to track how local age-friendliness changes as age-friendly city and community care policies are implemented. Second, the same 89 districts were used to refine the indicators and evaluate the final models. Therefore, the retained structure may be specific to the present sample. Further studies should examine whether the same structure is reproduced in a larger independent sample, the full population of si-gun-gu districts, or administrative data from other years. Until such validation is completed, the refined Index should be used as a preliminary diagnostic tool and not as the sole basis for municipal rankings, performance evaluation, or resource-allocation decisions. Third, the Index relied primarily on indicators calculated relative to the older population, which may not fully capture spatial accessibility. Although this approach reflects the availability of resources per older resident, it does not account for geographic area, settlement dispersion, travel distance, or transportation barriers. This is particularly relevant for rural areas, where resources may appear adequate on a per capita basis but remain difficult for older adults to reach in practice. Future research should incorporate area-based and spatial accessibility measures, such as resources per square kilometer, travel time to facilities, public transportation connectivity, or service catchment areas. Such measures would help distinguish between the nominal availability of age-friendly resources and their actual accessibility for older adults. Finally, the present study focused on the internal measurement structure of the Index. Therefore, although this analysis indicates whether the retained indicators function together within their intended dimensions, it does not establish whether the resulting scores reflect meaningful differences in older adults’ health, functioning, social participation, service use, or ability to remain in their communities. Consequently, future research should examine criterion, predictive, and external validity by testing associations between district-level age-friendliness scores and these relevant outcomes. The measurement structure should also be replicated using independent samples or administrative data from other years.

Despite these limitations, this study makes several important contributions. First, it provides empirical evidence regarding the internal measurement structure and refinement of an administrative-data-based Age-Friendly City Index at the municipal district level. Second, the refined Index offers a preliminary diagnostic framework for identifying relative strengths and weaknesses in local physical, social, and service environments. Third, by revealing regional disparities in age-friendliness, especially in the physical environment, the study highlights the need for region-specific public health and urban planning strategies. Overall, the refined index provides a more parsimonious and empirically grounded framework for assessing local age-friendliness.

## 5. Conclusions

This study empirically examined and refined an administrative-data-based Age-Friendly City Index for South Korean municipalities. The refinement process showed that conceptually relevant indicators do not necessarily form empirically coherent dimensions. The physical environment emerged as the most empirically cohesive dimension, whereas evidence supporting the social and service dimensions was more limited and should be confirmed in independent samples. Exploratory comparisons indicated substantial metropolitan–non-metropolitan differences in resource availability relative to the size of the older population, with the largest standardized difference observed in the physical environment. The refined Index therefore offers a preliminary diagnostic framework for identifying dimension-specific gaps and informing region-specific healthy ageing-in-place policies. However, criterion validity and measurement invariance should be established, and the refined structure should be replicated in larger independent samples before the Index is used as a basis for definitive municipal benchmarking or policy decisions.

## Figures and Tables

**Table 1:** Composition of the Age-Friendly City Index: original indicators, modifications, and calculation methods.

Dimension	Subdomain	Original Indicator	Calculation	Modification (Data Source)
Physical	Outdoor spaces and buildings	CCTV cameras	$\left(\frac{Number\;of\;CCTV\;cameras}{Number\;of\;older\;residents\;}\right)*1000$	Retained (Local Administration Integrated Information System of the Ministry of the Interior and Safety)
		Number of police-linked safety emergency bells	$\left(\frac{Number\;of\;police-linked\;safety\;emergency\;bells}{Number\;of\;older\;residents\;}\right)*1000$	Retained (Open Government Data Portal of the Ministry of the Interior and Safety)
		Air quality (fine dust concentration) ^a^	$\left(\frac{1}{Average\;fine\;dust\;concentration\;\left(PM2.5\;and\;PM10\right)}\right)*100$	Retained (AIRKOREA of the Korean Ministry of Environment and the Korea Environment Corporation)
		Street lights ^b^	$\left(\frac{Number\;of\;security\;lights}{Number\;of\;older\;residents\;}\right)*1000$	Replaced with security lights (Open Government Data Portal of the Ministry of the Interior and Safety)
		Number of silver zones	$\left(\frac{Number\;of\;silver\;zones}{Number\;of\;older\;residents\;}\right)*10,000$	Retained (Open Government Data Portal of the Ministry of the Interior and Safety)
		Park area	$\left(\frac{Park\;area\;in\;m^{2}}{Number\;of\;older\;residents\;}\right)$	Retained (Korean Statistical Information Service of Statistics Korea)
		Shade screens ^b^	$\left(\frac{Number\;of\;heat\;wave\;shelters}{Number\;of\;older\;residents\;}\right)*1000$	Replaced with heat-wave shelters (National Disaster and Safety Portal of the Ministry of the Interior and Safety)
		Public toilets	$\left(\frac{Number\;of\;public\;toilets}{Number\;of\;older\;residents\;}\right)*1000$	Retained (Local Administration Integrated Information System of the Ministry of the Interior and Safety)
	Transportation	Bus stops ^c^	$\left(\frac{Number\;of\;bus\;and\;subway\;stops}{Number\;of\;older\;residents\;}\right)*1000$	Expanded to include subway stops (Transportation Card Big Data Integrated Information System of the Ministry of Land, Infrastructure and Transport, and the Korea Transportation Safety Authority)
		Older adults injured or killed in traffic accidents ^d^	$\left(\frac{1}{Traffic\;accident\;rate\;among\;older\;adults\;per\;1000\;older\;residents}\right)$	Retained Traffic Accident Analysis System (TAAS of the Road Traffic Authority)
	Housing	Capacity of residential welfare facilities for older adults	$\left(\frac{Capacity\;of\;residential\;welfare\;facilities\;for\;older\;adults}{Number\;of\;older\;residents\;}\right)*1000$	Retained (e-country indicators of Statistics Korea)
		Number of public and private rental housing units	$\left(\frac{Number\;of\;public\;and\;private\;rental\;housing\;units}{Number\;of\;older\;residents\;}\right)*1000$	Retained (e-country indicators of Statistics Korea)
Social	Social participation	Leisure welfare facilities for older adults	$\left(\frac{Number\;of\;leisure\;welfare\;facilities\;for\;older\;adults}{Number\;of\;older\;residents\;}\right)*1000$	Retained (e-country indicators of Statistics Korea)
		Traditional markets	$\left(\frac{Number\;of\;traditional\;markets}{Number\;of\;older\;residents\;}\right)*10,000$	Retained (Open Government Data Portal of the Ministry of Interior and Safety)
	Social integration	Senior stores ^e^	$\left(\frac{\begin{array}{c} Number\;of\;organizations\;providing\;\\ employment\;programs\;for\;older\;adults \end{array}}{Number\;of\;older\;residents\;}\right)*10,000$	Replaced with organizations providing employment programs for older adults (Senior Job Yeogi of the Ministry of Health and Welfare, and the Korean Labor Force Development Institute for the Aged)
		Free food service centers for older adults	$\left(\frac{Number\;of\;free\;food\;service\;centers\;for\;older\;adults}{Number\;of\;older\;residents\;}\right)*10,000$	Retained (Open Government Data Portal of the Ministry of the Interior and Safety)
	Education and informatization	Lifelong educational institutions	$\left(\frac{Number\;of\;lifelong\;educational\;institutions}{Number\;of\;older\;residents\;}\right)*10,000$	Retained (Korea Education Statistics Service of Lifelong Education Statistics Survey conducted by the Ministry of Education and the Korea Educational Development Institute)
		Education programs for older adults	$\left(\frac{Number\;of\;educational\;programs\;for\;older\;adults}{Number\;of\;older\;residents\;}\right)*1000$	Retained (Korea Education Statistics Service of Lifelong Education Statistics Survey conducted by the Ministry of Education and the Korea Educational Development Institute)
Service	Health care services	Hospitals ^f^	$\left(\frac{Number\;of\;hospital\;beds}{Number\;of\;older\;residents\;}\right)*1000$	Replaced with hospital beds (Korean Statistical Information Service of Statistics Korea)
		Doctors	$\left(\frac{Number\;of\;doctors}{Number\;of\;older\;residents\;}\right)*1000$	Retained (Korean Statistical Information Service of Statistics Korea)
		Subjective health status ^g^	Self-rated health status of older adults (%)	Removed
		Health check-up rate ^g^	Health check-up rate among older adults (%)	Removed
		Strength and flexibility exercise rate ^g^	Strength and flexibility exercise rate among older adults (%)	Removed
		Rate of no suicidal ideation ^g^	Rate of older adults reporting no suicidal ideation (%)	Removed
	Welfare services	Medical welfare facilities for older adults	$\left(\frac{Number\;of\;medical\;welfare\;facilities\;for\;older\;adults}{Number\;of\;older\;residents\;}\right)*10,000$	Retained (e-country indicators of Statistics Korea)
		In-home welfare facilities for older adults	$\left(\frac{Number\;of\;in-home\;welfare\;facilities\;for\;older\;adults}{Number\;of\;older\;residents\;}\right)*10,000$	Retained (e-country indicators of Statistics Korea)
		Care service facilities for older adults	$\left(\frac{Number\;of\;care\;service\;facilities\;for\;older\;adults}{Number\;of\;older\;residents\;}\right)*10,000$	Retained (Open Government Data Portal of the Ministry of the Interior and Safety)

Note. All indicators are calculated at the district level, with both the numerator and denominator from the same district. ^a^ Computed as the inverse so higher values indicate better air quality. ^b^ Replaced because of limited district-level data. ^c^ Subway stops added to capture urban rail transportation. ^d^ Reverse-scored so fewer accidents indicate greater age-friendliness. ^e^ Broader measure of senior employment. ^f^ Hospital beds capture institutional capacity more fully than hospital counts. ^g^ Individual-level (person-attribute) indicators removed to reflect environmental conditions only.

**Table 2:** Model Fit Statistics for the Confirmatory Factor Analysis of Each Dimension at Successive Steps of Indicator Refinement (*N* = 89).

Dimension/Step	Indicators	$\chi^{2}$	df	RMSEA	CFI	TLI	SRMR
Physical							
Step 1. Initial	12	111.747	54	0.110	0.584	0.492	0.114
Step 2. Negative loadings removed	10	59.452	35	0.089	0.728	0.650	0.096
Step 3. Final	4	0.041	2	0.000	1.000	1.000	0.004
Social							
Step 1. Initial	7	57.812	14	0.189	0.637	0.456	0.124
Step 2. Final	3	0.000	0	0.000	1.000	1.000	0.000
Service							
Step 1. Initial	5	6.328	5	0.055	0.983	0.966	0.048
Step 2. Final	3	0.000	0	0.000	1.000	1.000	0.000

Note. All models were estimated with maximum likelihood with robust standard errors (MLR). RMSEA = Root Mean Square Error of Approximation; CFI = Comparative Fit Index; TLI = Tucker–Lewis Index; SRMR = Standardized Root Mean Square Residual.

**Table 3:** Factor Loadings for the Final Measurement Model of Each Dimension (*N* = 89).

Dimension/Indicator	B	SE	β	$R^{2}$
Physical				
Security lights	1.000	−	0.758 ***	0.574
Heat-wave shelters	0.040	0.009	0.768 ***	0.590
Public toilets	0.019	0.005	0.476 ***	0.226
Road-crossing safety	0.009	0.002	0.575 ***	0.331
Social				
Leisure welfare facilities	1.000	−	0.425 *	0.181
Traditional markets	0.130	0.087	0.356 *	0.127
Organizations providing employment programs	0.223	0.191	0.616 *	0.379
Service				
Medical welfare facilities	1.000	−	0.370	0.137
In-home welfare facilities	1.057	1.011	0.423	0.179
Care-service facilities	0.106	0.098	0.419	0.176

Note. B = unstandardized loading; β = standardized loading. The first indicator of each dimension was fixed to 1.0 for model identification. The first indicator of each dimension was fixed to 1.0 for model identification. * *p* < 0.05, *** *p* < 0.001.

**Table 4:** Descriptive Statistics for the Age-Friendliness Scores by Dimension (*N* = 89).

Dimension	M	SD	Min	Max
Physical	50.0	7.5	40.5	70.6
Social	50.0	6.9	39.5	66.9
Service	50.0	6.6	37.4	65.7
Total	150.0	17.6	124.3	193.8

**Table 5:** Comparison of age-friendliness scores between metropolitan and non-metropolitan districts.

Dimension	Metropolitan (*n* = 38) M (SD)	Non-Metropolitan (*n* = 51) M (SD)	Mean Difference [95% CI]	*t*	Cohen’s d
Physical	45.3 (4.5)	53.5 (7.3)	8.2 [5.7, 10.7]	6.54 ***	1.31
Social	47.7 (5.3)	51.7 (7.5)	3.9 [1.2, 6.6]	2.91 **	0.59
Service	46.4 (5.5)	52.7 (6.1)	6.4 [3.9, 8.8]	5.14 ***	1.08
Total	139.4 (11.5)	157.9 (17.2)	18.5 [12.5, 24.6]	6.07 ***	1.23

Note. The metropolitan group comprised municipalities in Seoul and the six metropolitan cities (*n* = 38), whereas the non-metropolitan group comprised municipalities in the eight provinces classified under the small-to-medium-city or rural-area categories in the sampling frame (*n* = 51). Mean differences were calculated as non-metropolitan minus metropolitan scores; positive values therefore indicate higher scores in non-metropolitan municipalities. Welch’s *t* tests were used as equal variances were not assumed. Mean differences with 95% confidence intervals and Cohen’s d were reported to indicate the magnitude and precision of the group differences. ** *p* < 0.01, *** *p* < 0.001.

## Data Availability

The data used in this study were derived from publicly available national administrative data sources. The data sources for each indicator are listed in Table 1.

## Abbreviations

The following abbreviations are used in this manuscript:

AVE	Average Variance Extracted
CFA	Confirmatory Factor Analysis
CR	Composite Reliability
FIML	Full-Information Maximum Likelihood
WHO	World Health Organization

## Appendix A

**Table A1:** Standardized factor loadings (β) at each step of indicator refinement for the three dimensions (*N* = 89).

Dimension/Indicator	Step 1	Step 2	Final
Physical	12 items	10 items	4 items
CCTV cameras	0.190	0.221	Removed
Safety bells	−0.340	Removed	Removed
Air quality	0.321	0.264	Removed
Security lights	0.712	0.718	0.758
Silver zones	0.013	0.014	Removed
Parks	0.132	0.202	Removed
Heat-wave shelters	0.821	0.782	0.768
Public toilets	0.478	0.511	0.476
Bus and subway stops	0.060	0.125	Removed
Road-crossing safety	0.558	0.582	0.575
Residential welfare facilities	0.172	0.219	Removed
Rental housing	−0.367	Removed	Removed
Social	7 items		3 items
Leisure welfare facilities	0.226		0.425
Traditional markets	−0.003		0.356
Employment-program organizations	0.104		0.616
Free meal service centers	0.009		Removed
Lifelong education institutions	−0.275		Removed
Education programs for older adults	−0.881		Removed
Lifelong education participants	−0.922		Removed
Service	5 items	n/a	3 items
Hospital beds	0.934		Removed
Doctors	0.762		Removed
Medical welfare facilities	−0.373		0.370
In-home welfare facilities	−0.052		0.423
Care-service facilities	−0.525		0.419

Note: n/a = not applicable, as the Social and Service dimensions underwent a two-step indicator selection process without an intermediate Step 2.

**Table A2:** District-Level Demographic Characteristics and Age-Friendliness Scores (*N* = 89).

Region	Number of Older Adults	Proportion of Older Adults (%)	Physical	Social	Service	Total
Gangwon-do						
1. Yangyang-gun	8009	28.7	62.2	58.9	65.5	186.6
2. Wonju-si	50,964	14.4	49.1	45.9	54.1	149.0
3. Chuncheon-si	48,291	17.1	47.8	51.0	61.3	160.1
4. Hongcheon-gun	17,113	24.7	56.2	48.1	54.3	158.5
Gyeonggi-do						
5. Goyang-si	138,561	12.8	44.2	41.3	52.9	138.4
6. Gwacheon-si	7801	12.3	51.3	53.1	50.8	155.2
7. Gwangju-si	48,508	12.7	48.0	40.1	44.0	132.0
8. Gimpo-si	54,482	11.5	46.5	42.4	47.4	136.3
9. Namyangju-si	95,717	13.4	46.6	39.5	59.2	145.4
10. Dongducheon-si	18,461	19.6	48.9	48.1	60.4	157.4
11. Bucheon-si	109,531	13.4	43.1	44.8	47.4	135.2
12. Seongnam-si	128,291	13.6	45.1	46.0	42.6	133.7
13. Ansan-si	73,140	11.2	48.5	41.9	60.7	151.1
14. Anseong-si	32,035	17.1	64.1	50.9	51.4	166.4
15. Anyang-si	73,617	13.4	44.3	44.4	47.3	136.0
16. Yongin-si	139,797	13.0	42.1	40.9	48.1	131.1
17. Uijeongbu-si	66,426	14.4	41.9	43.2	53.3	138.4
18. Pyeongtaek-si	61,663	11.5	51.0	47.6	48.9	147.4
19. Hanam-si	35,125	12.0	51.9	42.0	43.4	137.3
Gyeongsangnam-do						
20. Geochang-gun	16,197	26.3	70.6	60.3	57.5	188.4
21. Goseong-gun	15,187	29.6	62.9	62.4	52.3	177.6
22. Gimhae-si	59,032	10.9	47.7	48.6	46.6	142.9
23. Sacheon-si	23,662	21.3	63.4	66.1	55.1	184.6
24. Jinju-si	57,144	16.4	49.8	48.9	43.2	141.9
25. Changwon-si	144,919	14.0	48.8	57.5	44.8	151.2
26. Hadong-gun	14,493	32.4	57.1	62.6	52.7	172.4
Gyeongsangbuk-do						
27. Gyeongsan-si	43,458	16.5	48.0	44.9	52.0	145.0
28. Gumi-si	38,874	9.3	54.5	58.4	56.0	168.8
29. Yeongcheon-si	27,134	26.6	56.7	49.5	47.6	153.8
30. Cheongdo-gun	15,022	35.5	66.1	58.1	50.1	174.3
31. Chilgok-gun	17,280	15.1	60.9	45.7	55.4	162.1
32. Pohang-si	84,542	16.8	52.3	53.5	44.3	150.2
Gwangju						
33. Gwangsan-gu	38,455	9.5	56.5	52.9	59.5	169.0
34. Nam-gu	37,715	17.6	49.1	53.2	55.7	158.0
35. Buk-gu	64,829	15.1	48.4	48.5	52.4	149.4
Daegu						
36. Dalseo-gu	78,619	14.1	46.6	50.6	46.8	144.0
37. Dong-gu	65,108	19	50.9	45.0	48.5	144.4
38. Buk-gu	62,033	14.1	49.0	50.8	48.3	148.1
39. Seo-gu	37,656	22.1	49.5	58.2	54.7	162.4
Daejeon						
40. Seo-gu	59,000	12.3	48.0	45.0	52.5	145.4
41. Yuseong-gu	33,000	9.4	56.6	49.3	48.4	154.3
42. Jung-gu	45,000	19.1	45.7	49.8	50.3	145.8
Busan						
43. Nam-gu	52,411	19.6	41.5	47.4	41.7	130.6
44. Buk-gu	46,810	16.5	40.9	45.9	46.9	133.7
45. Saha-gu	57,188	18.3	45.6	49.8	44.2	139.6
46. Seo-gu	25,420	23.5	46.5	62.2	42.0	150.8
47. Jung-gu	10,470	25.2	53.9	58.1	47.8	159.7
48. Busanjin-gu	66,404	18.5	43.9	55.2	37.6	136.7
Seoul						
49. Gangnam-gu	69,057	12.8	46.4	45.2	37.4	129.0
50. Gangdong-gu	62,599	13.6	42.4	41.7	44.1	128.2
51. Gangbuk-gu	59,706	19.4	41.0	45.1	45.5	131.5
52. Gangseo-gu	85,069	14.7	41.1	45.4	42.8	129.3
53. Gwanak-gu	75,763	15.3	41.7	44.7	44.4	130.8
54. Guro-gu	70,722	17.5	41.9	44.2	40.7	126.9
55. Geumcheon-gu	40,064	17.3	43.0	43.5	47.6	134.1
56. Nowon-gu	81,044	15.5	41.3	41.6	42.9	125.9
57. Dongdaemun-gu	58,353	17.0	45.5	47.9	45.5	138.9
58. Dongjak-gu	62,631	16.0	43.4	45.5	40.1	129.1
59. Mapo-gu	50,138	13.5	43.4	52.4	40.8	136.5
60. Seocho-gu	55,628	13.1	46.4	39.8	38.1	124.3
61. Seongbuk-gu	68,903	15.8	43.3	41.9	41.4	126.5
62. Yangcheon-gu	61,741	13.6	42.7	48.1	52.5	143.4
63. Eunpyeong-gu	79,653	16.6	41.3	42.8	41.1	125.2
Ulsan						
64. Nam-gu	39,264	12.3	53.9	52.1	43.7	149.7
65. Dong-gu	20,002	12.8	45.1	53.9	46.3	145.4
Incheon						
66. Namdong-gu	69,924	13.3	41.8	42.6	50.7	135.2
67. Michuhol-gu	67,284	16.6	40.5	42.8	45.0	128.4
68. Bupyeong-gu	71,857	14.5	41.0	44.3	46.9	132.2
69. Seo-gu	56,595	10.4	40.6	42.9	59.0	142.6
70. Yeonsu-gu	35,941	9.3	40.6	43.8	47.6	132.1
Jeollanam-do						
71. Gwangyang-si	19,228	12.7	48.3	58.2	54.0	160.5
72. Damyang-gun	13,370	28.9	66.9	55.1	59.3	181.2
73. Mokpo-si	37,599	16.8	54.2	52.7	59.1	166.0
74. Yeonggwang-gun	14,349	27.0	61.2	66.9	65.7	193.8
75. Hwasun-gun	15,643	25.0	67.7	57.8	61.3	186.9
Jeollabuk-do						
76. Gochang-gun	17,865	32.8	49.7	65.4	54.6	169.8
77. Gunsan-si	47,873	17.9	54.4	52.9	54.5	161.8
78. Wanju-gun	20,604	22.5	61.0	62.8	64.1	187.9
79. Iksan-si	53,522	19.0	51.4	56.9	56.9	165.3
80. Jeonju-si	97,039	14.8	45.3	46.7	51.2	143.2
Chungcheongnam-do						
81. Gongju-si	26,393	25.2	54.3	53.6	55.0	163.0
82. Nonsan-si	29,835	25.6	51.7	54.1	45.6	151.4
83. Dangjin-si	29,963	18.0	58.1	48.9	46.0	153.0
84. Asan-si	41,295	13.1	52.6	45.5	51.2	149.2
85. Cheonan-si	70,488	10.7	51.0	47.6	50.7	149.2
Chungcheongbuk-do						
86. Okcheon-gun	14,400	28.5	55.2	56.5	50.8	162.4
87. Jeungpyeong-gun	6200	16.8	64.7	64.0	62.5	191.2
88. Cheongju-si	109,200	12.9	51.9	49.1	49.1	150.1
89. Chungju-si	40,500	19.3	58.4	54.1	46.5	159.0
